# Epidemiology of the avian influenza A (H7N9) outbreak in Zhejiang Province, China

**DOI:** 10.1186/1471-2334-14-244

**Published:** 2014-05-08

**Authors:** Zhenyu Gong, Huakun Lv, Hua Ding, Jiankang Han, Jimin Sun, Chengliang Chai, Jian Cai, Zhao Yu, Enfu Chen

**Affiliations:** 1 Zhejiang Provincial Center for Disease Control and Prevention, Hangzhou, China; 2 Hangzhou Municipal Center for Disease Control and Prevention, Hangzhou, China; 3 Huzhou Municipal Center for Disease Control and Prevention, Huzhou, China

**Keywords:** Avian influenza A (H7N9) virus, Epidemiology, Rural cases, Multi-exposure

## Abstract

**Background:**

A novel influenza A virus infection was identified on March 31, 2013 in China and a total of 134 cases were identified in 12 provinces of China between March 25 and September 31, 2013. Of these, 46 cases occurred in Zhejiang Province and the number of patients is the largest in China.

**Methods:**

Field investigations were conducted for each confirmed H7N9 case. A standardized questionnaire was used to collect information about demographics, exposure history, clinical signs and symptoms, timelines of medical visits and care after onset of illness, and close contacts. Descriptive statistics were used to analyze the epidemiological and clinical characteristics. Samples from the patients were collected and tested by real time reverse transcriptase-polymerase chain reaction and viral culture.

**Results:**

A total of 46 laboratory confirmed cases of H7N9 influenza infection were identified in the Zhejiang province between March 31 and September 31, 2013 of which 29 were male and 17 were female. The median age of patients was 61.5 years and 76.09% of cases occurred in persons aged ≥50 years old. Unlike other province, 34.78% of cases in Zhejiang Province were rural residents. Among 11 deaths, 9 were male, 10 were older than 60 years old, and 10 had underlying diseases. 30 of 38 cases with available data had a recent history of poultry exposures and 8 cases had multi-exposure history. The estimated median incubation period was two days which was shorter than corresponding data in other provinces. All cases were hospitalized and the median time from illness onset to hospitalization was 5 days. Symptoms at the onset of the illness included fever, cough, expectoration, shivering, fatigue, muscular aches, nausea, vomiting. Only 4.91% contacts developed respiratory symptoms, but their samples were tested negative for H7N9 virus designating lack of human-to-human transmission of the virus.

**Conclusions:**

All cases were sporadic and there was no evidence of an epidemiologic link between them. Control measures including closing affected poultry and slaughtering backyard poultry are needed not only in urban areas but also in rural areas to reduce human H7N9 infection risk.

## Background

Type A influenza viruses are classified into 16 hemagglutinin (HA) subtypes and 9 neuraminidase (NA) subtypes [1]. Influenza A viruses are further divided into low-pathogenic avian influenza (LPAI) and high-pathogenic avian influenza (HPAI) viruses based on their pathogenic properties in chickens. Fowl were infected with avian influenza A (H7) viruses in Italy in 2000, Chile in 2002, the Netherlands in 2003, British Columbia, Canada in 2004, and Saskatchewan, Canada in 2007 [2]. Human infections with H7 influenza viruses (H7N2, H7N3, and H7N7) were reported in the Netherlands, Italy, Canada, United States of America, Mexico and the United Kingdom [3-5]. Most A (H7) infections in humans were mild except one death during a large outbreak in the Netherlands involving highly pathogenic A(H7N7) [6,7].

On March 31 the Chinese Authorities (the National Health and Family Planning Commission, previously the Ministry of Health) announced the identification of a novel influenza A virus infection, A (H7N9) in three people who were seriously ill from two Chinese provinces [8]. This is the first time that human infection with influenza A (H7N9) virus has been identified. The virus is an avian influenza A reassortant of A (H7N9) from which the haemagglutinin and the neuraminidase genes have their originate, and A (H9N2) from which the other six gene segments are derived [9,10].

A total of 134 cases of H7N9 influenza infection were identified in 12 provinces of China between March 25 and September 31, 2013. Of these, 46 cases occurred in Zhejiang Province and the number of patients is the largest in China. In this study, we summarize the characteristics of this emerging infectious disease in Zhejiang Province.

## Methods

### Case definition

According to ‘the diagnosis and treatment programs of human infections with H7N9 virus’ issued by Chinese ministry of health [11], a suspected case is defined as a patient with influenza-like illness including fever, dry cough, headache, muscle ache, generalized malaise, and either positive laboratory confirmation of an influenza A virus, or recent history of exposure to poultry within one week before the onset of symptoms. We considered a suspected case to be a confirmed case if the H7N9 virus was isolated or H7N9 virus RNA was detected by real-time reverse transcription polymerase chain reaction (rRT-PCR) from respiratory specimens of the patient.

### Contacts definition

Close contacts were defined as medical staff or family members of a patient who met 1 or more of the following criteria: (1) did not take protective measures during diagnosis or treatment of suspected or confirmed cases or took care of the patient; (2) lived together or had history of close contact with the suspected or confirmed cases within the one week before the onset of illness; (3) were determined as close contacts by the investigators [12].

### Laboratory test assays

RNA was extracted from specimens with the use of the QIAamp Viral RNA Mini Kit (Qiagen) according to the manufacturer’s instructions and tested by rRT-PCR with H7N9-specific primers and probes as described previously [9]. These assays were carried out in biosafety level (BSL) 3 facilities at Zhejiang provincial CDC. Respiratory specimens were inoculated in amniotic cavities of pathogen-free embryonated chicken eggs for viral isolation.

### Data collection and analysis

A standardized questionnaire was used to collect information about demographic features, exposure history, clinical signs and symptoms, date of onset, date of first medical visit, number of medical visits after onset, date of hospitalization, date of specimen collection, date of viral infection confirmation and information on close contacts. Exposure history included the dates, times, frequency and patterns of exposures to poultry or other animals such as swine and wild birds during the two weeks before the onset of illness. Written informed consent for participation in the study was obtained from participants or their family members. An ethics waiver was granted and authorised under National Emergent Public Health Events Act. The investigation was exempt from institutional board assessment.

Descriptive statistics were used to analyze the epidemiologic characteristics and clinical characteristics of H7N9 cases in Zhejiang Province, China. Logistic regression analysis of fatality rate among gender, age groups, date groups of first medical visit, groups of time interval from illness onset to confirmation, groups of underlying diseases was conducted using the SPSS, version 16.0 statistical package (Chicago, IL, USA). The dependent variable in the logistic regression was assigned as the outcome of patients and the independent variables were gender, age group, underlying medical background, date group of first medical visit, group of time interval from illness onset to confirmation, and date group of first medical care × group of time interval from illness onset to confirmation. The method of logistic regression used was forward-conditional. The stepwise probability was set to 0.05 for entry and 0.10 for removal. The classification cutoff was 0.5 and the maximum number of iterations was 20.

## Results and Discussion

### Epidemiologic characteristics

**Figure 1:**
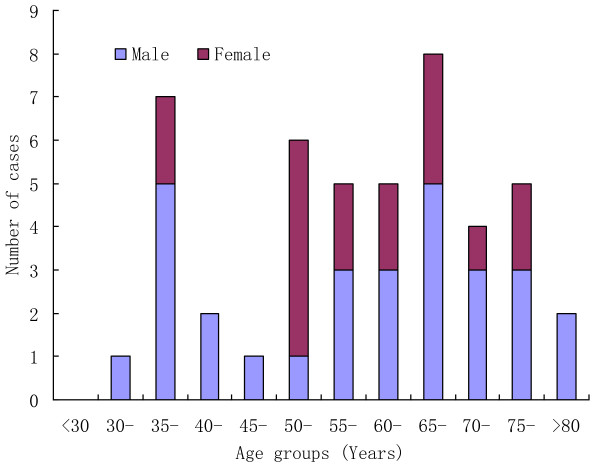
Age and gender distribution of H7N9 cases in Zhejiang Province.

**Figure 2:**
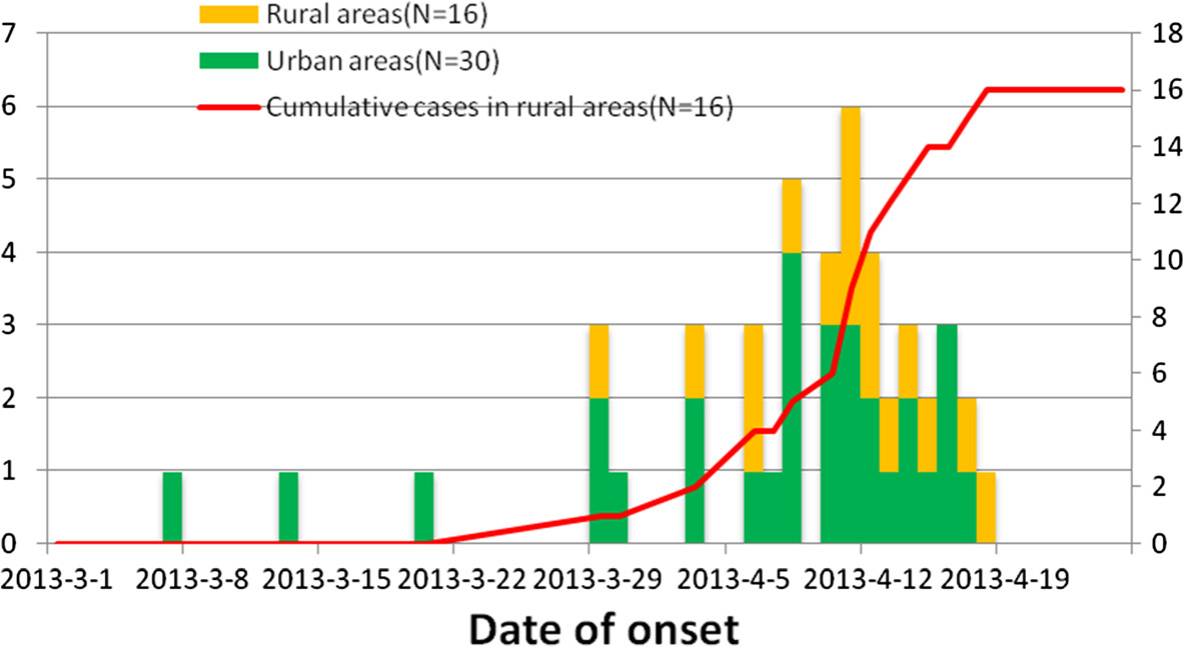
Timelines of H7N9 cases in urban and rural areas.

From March 25 to September 31, 2013, a total of 46 laboratory confirmed cases of H7N9 influenza infection were identified in the Zhejiang province of China of which 29 were male and 17 were female. Cases peaked in middle April and subsided in late April. The median age of confirmed patients was 61.5 years (range, 32–86 years) and 35 (76.09%) occurred in persons aged ≥50 years old (Figure 1). Initially, most cases were retired personnel who worked in urban areas and then the proportion of patients from rural areas increased (Figure 2). The proportion of rural residents in H7N9 cases from Zhejiang Province was 34.78% (16/46) which was higher than that of other provinces. Furthermore, one case was pregnant women and another case was government worker who attended slaughtering poultry in Huzhou city.

**Figure 3:**
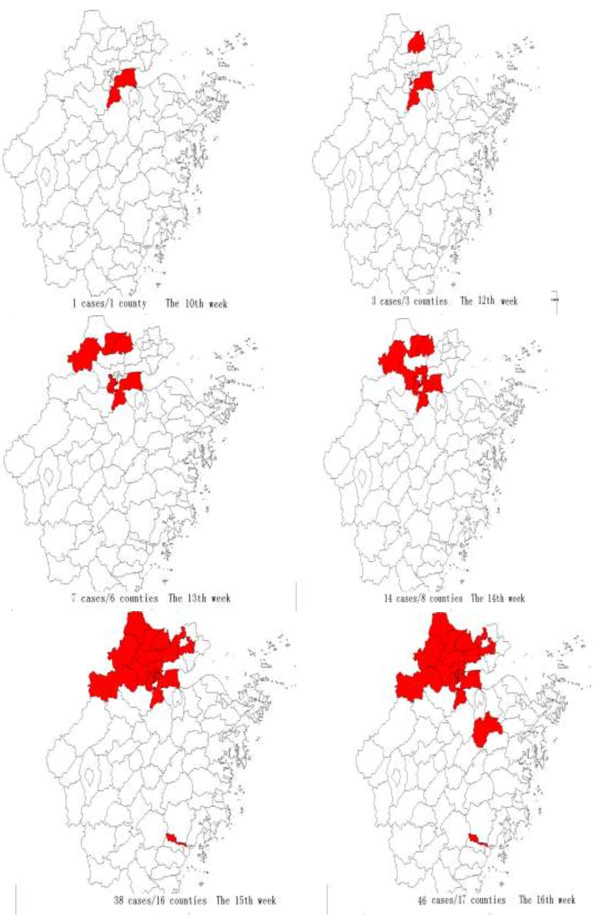
Geographical distribution of H7N9 cases in Zhejiang Province.

The first case had onset of symptoms on March 7 and was confirmed on April 3. Then the number of cases rose and peaked on April 11 (Figure 2). 39 (84.78%) cases had onset of symptoms after April 3. All cases occurred in 17 counties (Shangcheng, eight cases; Xiaoshan, six cases; Jianggan five cases; Xiacheng, four cases; Xihu, three cases; Yuhang, two cases; Gongshu, one case; Linan, one case; Wuxing, three cases; Nanxun, three cases; Anji, two cases; Changxing, two cases; Deqing, one case; Xiuzhou, one case; Tongxiang, one case; Lucheng, one case; and Shengzhou, one case), which were part of five cities. Cases occurred in more and more counties from the 10th week to 16th week, 2013 (Figure 3). The number of confirmed cases in Hangzhou city was the largest where the first case of Zhejiang Province was confirmed. Among 11 deaths, seven were male, 10 were older than 60 years old, and 10 had underlying medical background. According to results of logistic analysis, the Chi-square value in omnibus tests of model coefficients was determined to be 5.827 (P = 0.016 < 0.05). Variable in the equation was underlying medical background and the wald of it were determined to be 3.020. The equation was P = −5.774 + 1.699 × underlying medical background/(1 + Exp(−5.774 + 1.699 × underlying medical background)).

Among the 46 cases, 30 out of 38 (78.95%) with available data had a history of recent poultry exposures. Of the 30 cases with known poultry exposures, the type of poultry exposure inclued chickens, ducks, pigeons and bird, as shown in Table 1. Most cases (86.67%) were exposed to within the seven days before the onset of illness. Types of exposure to poultry included buying poultry (43.33%), visiting a poultry market (20.00%), breeding poultry (33.33%) and killing poultry (16.67%). Of interesting, eight cases had multi-exposure history (Table 2). Five cases bred backyard chicken and had exposure history of poultry markets, two cases had exposure history of different poultry markets, one case bred birds in the neighborhood and had exposure history of poultry markets. The estimated median incubation period for 30 confirmed cases with exposure history was two days.

**Table 1:** Exposure characteristic of 46 confirmed cases from Zhejiang Province

**Characteristic**	**Confirmed cases (n = 46)**
No exposure (%)	8/38 (21.05)
Poultry exposure history (%)	30/38 (78.95)
Chickens (%)	23/30(76.67)
Ducks (%)	6/30 (20.00)
Pigeons (%)	3/30 (10.00)
Bird (%)	1/30 (3.33)
Exposure frequency	
Every day before onset (%)	11(36.67)
<3 days before onset (%)	6 (20.00)
3-7 days before onset (%)	9 (30.00)
>7 days before onset (%)	4 (13.33)
Exposure ways	
Buying poultry (%)	13 (43.33)
Visiting poultry market (%)	6 (20.00)
Breeding poultry (%)	10 (33.33)
Killing and cleaning poultry (%)	5 (16.67)

**Table 2:** Multi-exposure history of some H7N9 cases

	**Exposure ways**	**Cases (n)**
Breeding chicken at home	Visiting poultry markets	2
	Selling birds at poultry markets	1
	Buying birds from poultry markets	2
Exposure to A poultry market	Exposure to B poultry market	2
	Breeding birds in the neighborhood	1
Total		8

There were 1038 close contacts of 46 confirmed cases and all contacts had been followed up for seven days. 51 (4.91%) contacts of 14 confirmed cases developed respiratory symptoms during the 7-day surveillance period. 528 throat swabs and 887 blood samples from close contacts were collected and tested negative for H7N9 via real-time RT-PCR. The median number of close contacts was 15.50 (range 3–125).

All cases were hospitalized and the median time interval from illness onset to hospitalization was five days (Table 3). Symptoms of the illness included fever (100.00%), cough (80.43%), expectoration (63.04%), shivering (26.09%), fatigue (28.26%), muscular aches (34.78%), nausea (6.52%), vomiting (4.35%). Among 38 confirmed cases with available data, 76.32% had underlying diseases including hypertension (62.07%), diabetes (31.03%), heart diseases (27.59%), trachitis (13.79%), and hepatitis (6.90%).

**Table 3:** Clinical characteristic of 46 confirmed cases from Zhejiang Province

**Clinical characteristics and timelines**	**Overall (n = 46)**
Clinical outcome	
Hospitalization (%)	46 (100)
Death (%)	11 (23.91)
Symptoms at the onset	
Fever (%)	46 (100.00)
Cough (%)	37 (80.43)
Expectoration (%)	29 (63.04)
Shivering (%)	12 (26.09)
Fatigue (%)	13 (28.26)
Muscular aches (%)	16 (34.78)
Nausea (%)	3 (6.52)
Vomiting (%)	2 (4.35)
Underlying diseases (%)	29/38 (76.32)
Hypertension (%)	18/29 (62.07)
Diabetes (%)	9/29 (31.03)
Heart diseases(%)	8/29(27.59)
Hepatitis (%)	2/29 (6.90)
Trachitis (%)	4/29 (13.79)
Timeline and duration, median, day (IQR)	
Illness onset to first medical care	1 (2.125)
Illness onset to hospitalization	5 (3.00)
Illness onset to specimen collection	6 (2.25)
Illness onset to confirmation	7 .5(3.00)
The number of medical visits	3 (2)

**Figure 4:**
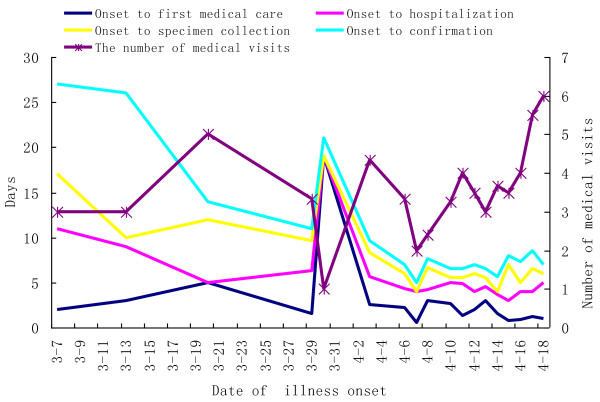
Timeline and frequency of medical care of H7N9 cases in Zhejiang Province.

The duration from illness onset to first medical visit of 46 confirmed cases was one day (range, 0–19 days). Specimen were collected a median of six days (range, 1–19 days) and cases were confirmed a median 7.5 days (range, 3–27 days) after illness onset. Time interval from illness onset to first medical visit, hospitalization, specimen collection, confirmation of cases decreased except those of one case whose time interval from illness onset to first medical care was 19 days (Figure 4).

The median number of medical visits of 46 confirmed cases was three (range, 1–6) times and 78.26% (36/46) patients visited hospital or an outpatient center at least three times. Surprisingly, the number of medical visits of latest cases rose slightly (Figure 4).

In our study, 46 cases were identified and most of them were severely ill patients including 11 deaths. All cases were sporadic and there was no evidence of an epidemiologic link between them. We suspect that these confirmed cases only represent the tip of the iceberg and that there are many more as-yet-undetected mild and symptomatic infections. The majority of cases were male and occurred among persons 50 years of age or older. It is different from that of H5N1 infection which occurred mainly in persons under 40 years of age [13,14]. The high proportion of elderly patients may be due to unbalanced exposure to poultry or may be due to physiological factors related to ageing, such as decreased immune function.

Another study has reported that 84% (69/82) of patients with H7N9 are urban residents [15]. In contrast, more than 1/3 of cases occurred in rural areas in our study. The reason behind this may be the acceleration of the integration of urban and rural areas in Zhejiang Province. As a result, live poultry entered rural areas shortly after their transactions were suspended in urban areas. Another reason may be that poultry in rural areas had been infected with the novel H7N9 virus via birds. However, we can’t explain whether cases in rural areas acquired their infections from live poultry markets or backyard poultry. H7N9 virus was transmitted in poultry markets in urban areas and no further H7N9 cases were identified after the affected markets were closed. However, H7N9 virus could have been transmitted among backyard poultry in rural areas. Not only were affected poultry markets closed, but it was recommended that backyard poultry should also be slaughtered.

The overall case fatality rate was 23.91% in our study which was lower than corresponding data of reported H5N1 virus infection [13,16-18]. The fatality rate may decrease as more and more mild cases were identified. Underlying medical background was a significant determinant of fatality rate. Similarly, chronic diseases were also associated with severe cases and deaths from seasonal influenza and H5N1 avian influenza [19,20]. No poultry outbreaks of H7N9 infection were identified in Zhejiang Province, 78.95% of cases with available data occurred in patients who had exposure to live poultry including contacts with chickens, ducks, pigeons and birds. The novel reassortant avian influenza virus may be of low pathogenicity to birds but of significant pathogenicity to humans. Interestingly, many cases didn’t have exposure history, many cases only had history of visiting live poultry markets and many cases had multi-exposure history. They may be infected via exposure to environments that were contaminated with infected poultry which was similar to infection route of H5N1 virus [21-23]. The number of new cases declined after control measures such as a ban on the selling of live poultry in market stalls or even market closure, poultry culling, and market disinfection were done indicating that infected poultry and contaminated environments may be infection sources. Furthermore, greater than 6% among 396 poultry workers were positive for antibodies specific for avian-origin H7N9 virus and the viral isolate from one patient in Zhejiang Province was closely similar to that from an epidemiological linked market chicken, confirming that infected poultry is the principal source of human infections too [24,25]. The incubation period of the H7N9 virus is generally less than seven days and the estimated median incubation period in our study was two days which was much shorter than corresponding data in the report of Liqun et al. [15]. Multi-exposure history may associated with the shorter incubation period.

Although 4.91% of contacts from 14 confirmed cases developed respiratory symptoms during the 7-day surveillance period, but specimen from them were tested negative for H7N9 via real-time RT-PCR. Follow-up prospective investigations of close contacts of patients with confirmed H7N9 virus infection had not conclusively established human-to-human H7N9 transmission to date. This indicated there was no other evidence pointing toward sustained transmission among people, which was different from H5N1 limited person-to-person transmission [26-28]. But we shouldn’t rule out limited human-to-human transmission, which was observed in the H7 outbreak in the Netherlands in 2003 and two family clusters of H7N9 infection were reported [7,15]. What’s more, another study suggested that avian influenza A (H7N9) virus was able to transmit from person to person according to epidemiological investigation although the transmissibility was limited and non-sustainable [29]. The median time interval from the onset of illness to hospitalization was five days, which echo that of a recent study [15] and was shorter than corresponding median times among patients with H5N1 virus infection [30]. The median time interval from the onset of illness to confirmation was 7.5 days and the majority of cases went to hospital at least three times implying that many cases delayed the golden period for treatment and increased severity of the disease.

Most of patients presented with flu-like symptoms, such as fever, and cough with little phlegm, which can be accompanied by headache, muscle aches, and general malaise. The clinical characteristics were similar with H7N9 cases in other provinces [31,32]. The majority of cases had underlying medical background suggested that co-morbidities may be a factor of H7N9 infection [33].

## Conclusions

As human infections with avian influenza A H7N9 virus are emerging, there is limited information about them. In our study, we summarized the epidemiologic characteristics and clinical characteristics of H7N9 infection in Zhejiang Province, such as the spatial and temporal distribution, probable infection source, close contacts, clinical outcome, symptoms at the onset, risk factors, and so on. These results provided baseline data for its control and prevention. Some results of our study were similar to the results of Liqun, but some results were significantly different with their results. For example, the proportion of rural residents was significantly higher in Zhejiang Province which informed that we should also pay attention to rural areas for H7N9 infection control and prevention. The incubation period in our study was much shorter than corresponding period in the study of Liqun as a result of multi-exposure history. Additionally, we analyzed risk factors for deaths which indicated that the underlying medical background had a strongly relationship with death due to H7N9 infection.

## Competing interests

No conflict of interest exits in the submission of this manuscript.

## Pre-publication history

The pre-publication history for this paper can be accessed here:

http://www.biomedcentral.com/1471-2334/14/244/prepub
